# Supplementary figures and images for: Identification of GGC Repeat Expansions in ZFHX3 Among Chilean Movement Disorder Patients
Source: medRxiv. 2025 Mar 19:2025.03.17.25323863. Preprint. [Version 1] doi: 10.1101/2025.03.17.25323863 (PMC11957069; doi:10.1101/2025.03.17.25323863)

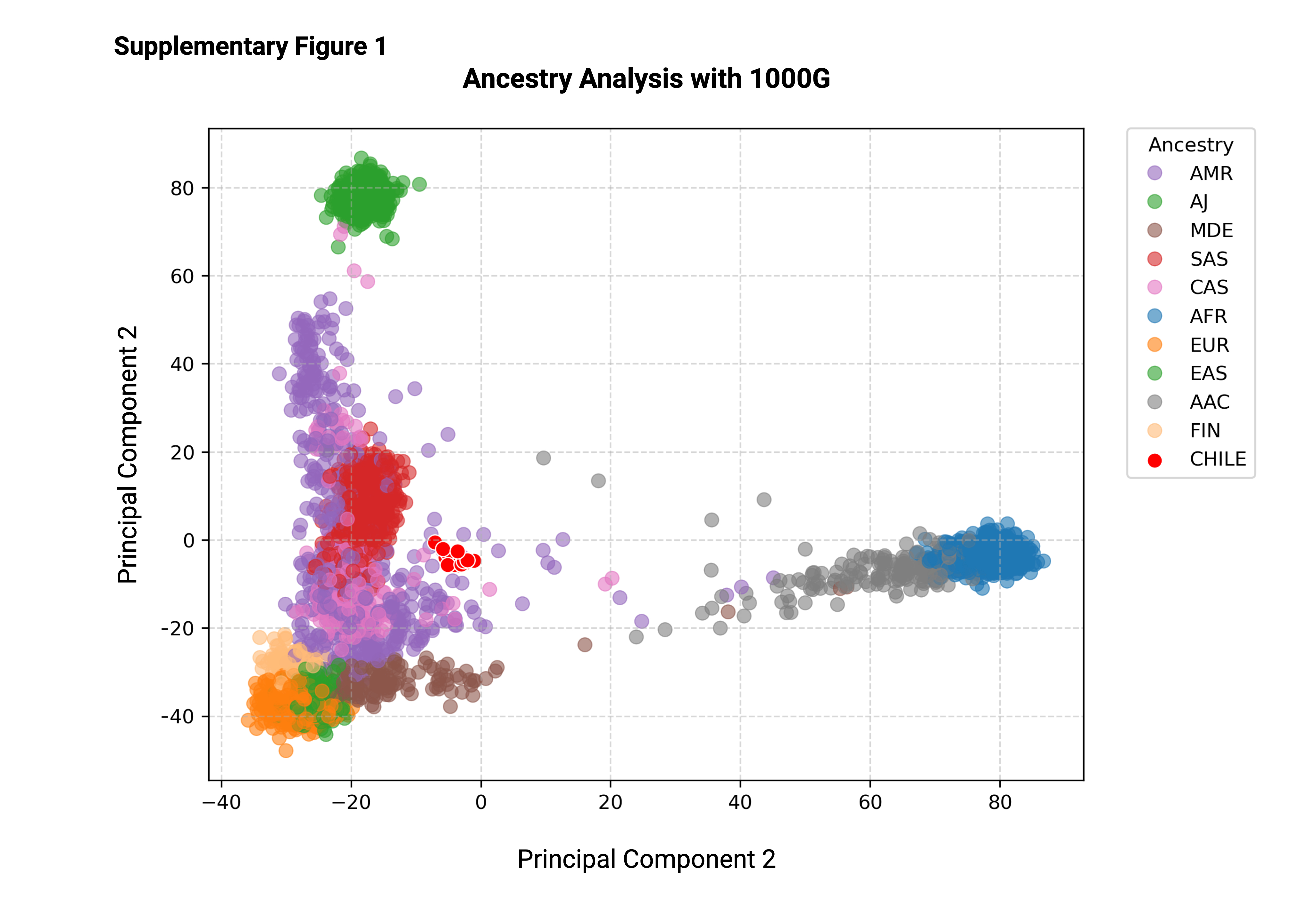

Supplement: Supplement 1 — Supplementary Figure 1. Scatter plot of principal components 1 and 2 from the Ancestry analysis Cluster plot of PC1 and PC2 of the ancestry analysis showed the Chilean samples clustering close to Latino/admixed American (AMR). AAC; African American/Afro-Caribbean, AFR; African, AJ; Ashkenazi Jewish, AMR; Admixed American, CAS; Central Asian, EAS; Eastern Asian, EUR; European, FIN; Finnish, MDE; Middle Eastern, SAS; South Asian. [file media-1.jpg]

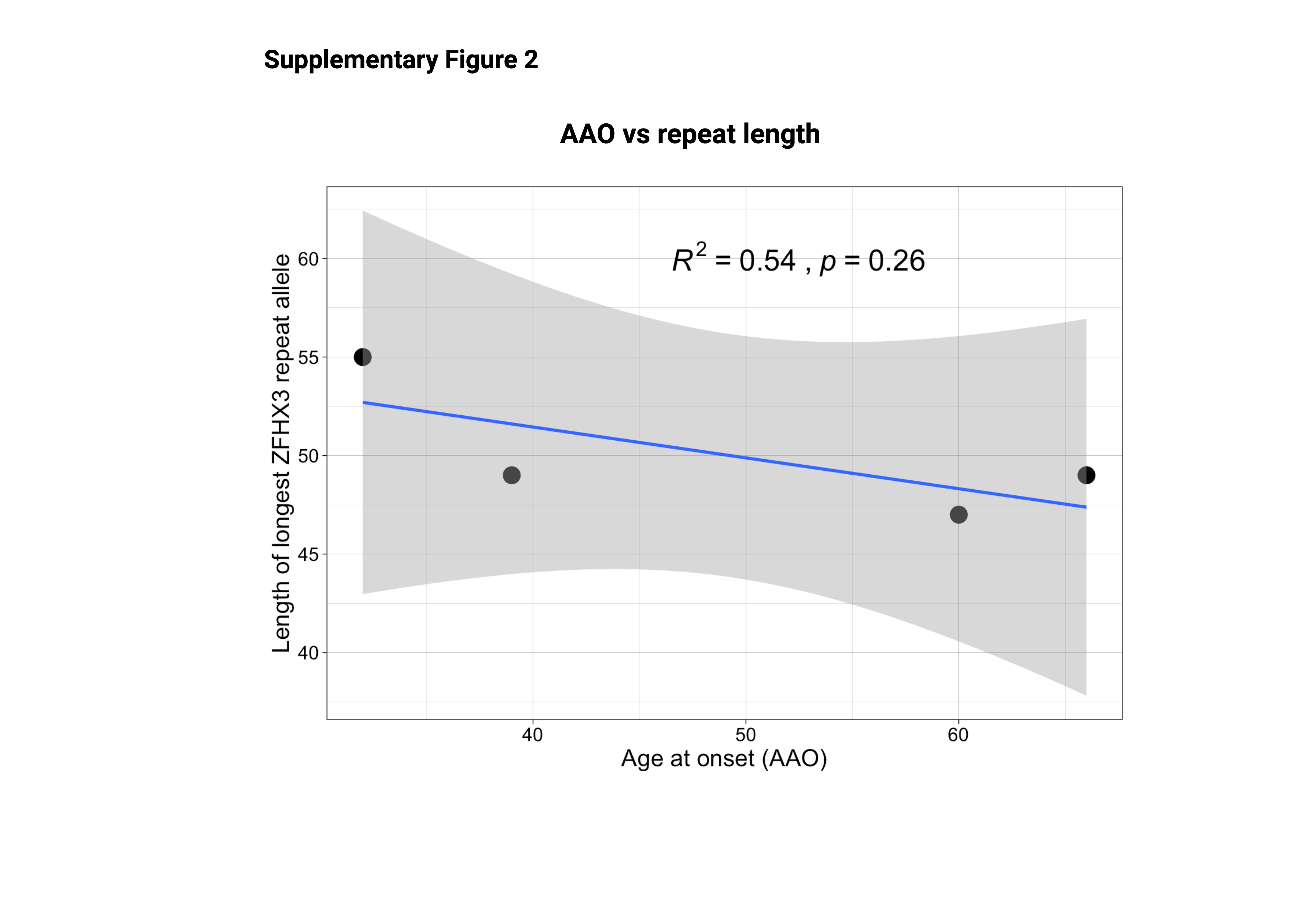

Supplement: Supplement 2 — Supplementary figure 2. Inverse Correlation Between ZFHX3 GGC Repeat Length and Age at Onset in SCA4 Patients. A negative trend is observed, but it is not statistically significant (R2 = 0.54, p = 0.26). [file media-2.jpg]
